# Supplementary figures and images for: Overexpression of AmCBF1 enhances drought and cold stress tolerance, and improves photosynthesis in transgenic cotton
Source: PeerJ. 2022 May 25;10:e13422. doi: 10.7717/peerj.13422 (PMC9147321; doi:10.7717/peerj.13422)

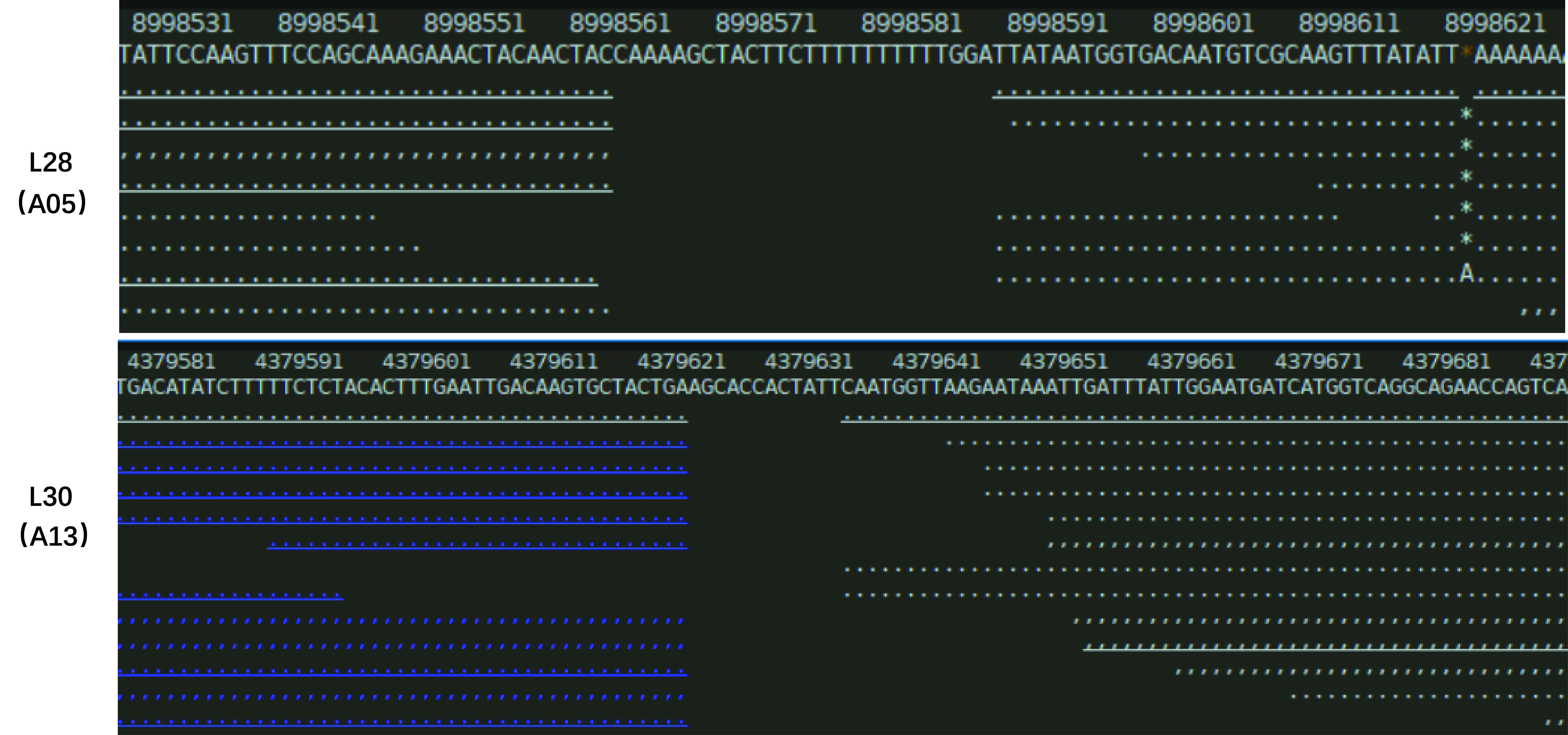

Supplement: Supplemental Information 1 — The whole-genome resequencing showed that insertion sites of L28 and L30 were located at 8998563–8998590 on chromosome A05 and 4379624–4379637 in A13 chromosome, respectively. [file peerj-10-13422-s001.jpg]

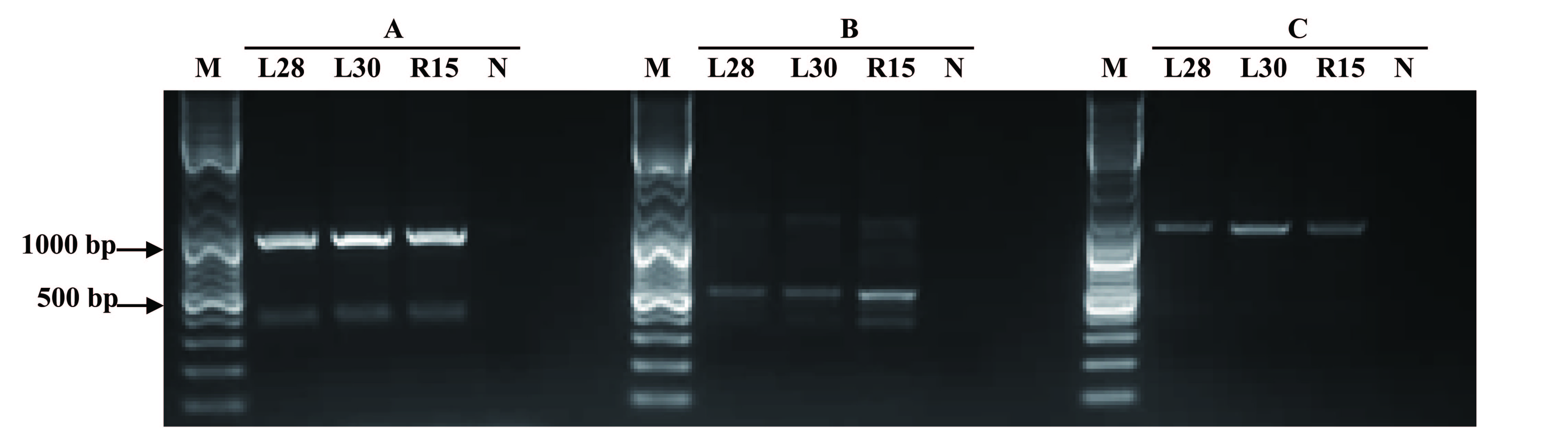

Supplement: Supplemental Information 2 — Three pairs of primers amplify the same size products in R15 and transgenic plants with the same sequence. A, Agarose gel of PCR products using primers A12-1F and A12-1R. B, Agarose gel of PCR products using primers A12-2F and A12-2R. C, Agarose gel of PCR products using primers A12-3F and A12-3R. Lane M, DNA marker; L28, transgenic cotton L28; L30, transgenic cotton L30; R15, wild type. N, negative control. [file peerj-10-13422-s002.jpg]

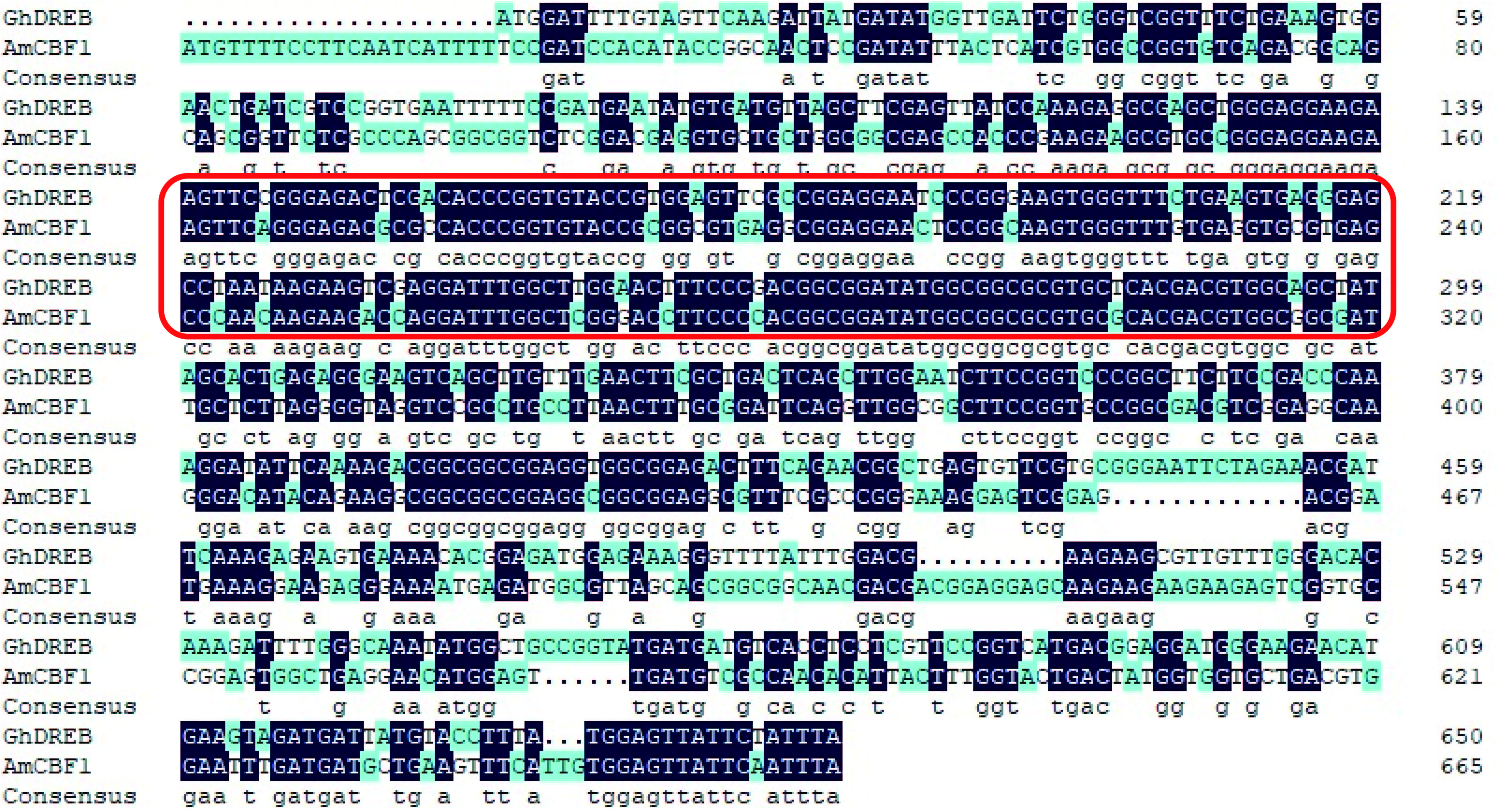

Supplement: Supplemental Information 3 — Sequencing analysis of the endogenous Gh DREB and AmCBF1 gene. The homology between the mRNA sequence of this GhDREB with AmCBF1 was 57.37%, The homology of the partial zone of this GhDREB with AmCBF1 (in the red box) reached 77.08%. [file peerj-10-13422-s003.jpg]

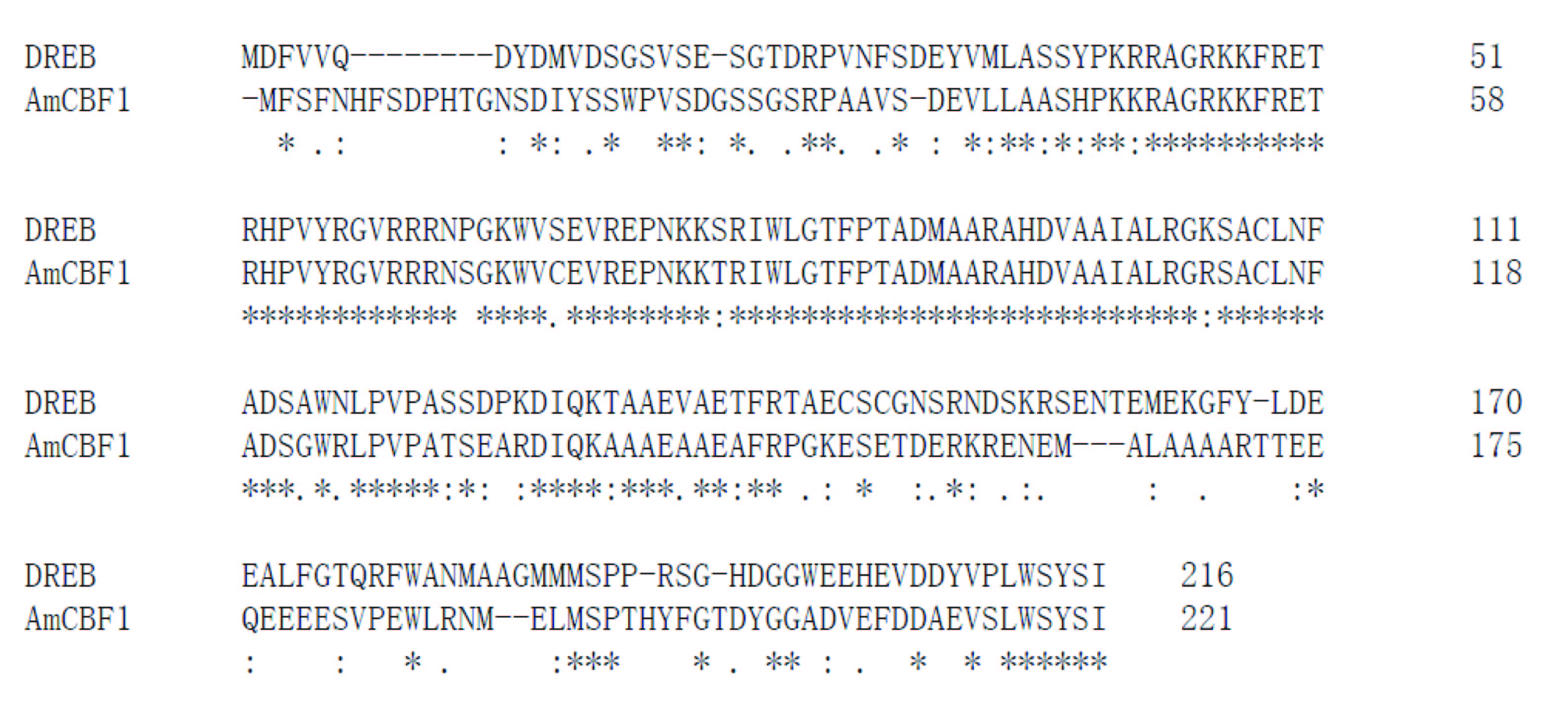

Supplement: Supplemental Information 4 — The amino acid sequence homology of the of GhDREB on A12 chromosome with AmCBF1 was 58.56%. [file peerj-10-13422-s004.jpg]
