# Supplementary material for: Overexpression of AmCBF1 enhances drought and cold stress tolerance, and improves photosynthesis in transgenic cotton
Source: PeerJ. 2022 May 25;10:e13422. doi: 10.7717/peerj.13422 (PMC9147321; doi:10.7717/peerj.13422)
Supplement: Supplemental Information 5 [file peerj-10-13422-s005.docx]

**Supplementary table 1** Primers used in this study

| **Primer purpose** | **Primer name** | **Primer sequence (5' to 3')** |
| --- | --- | --- |
| Specific primers for *AmCBF1* | *AmCBF1*-F | ACTATCCTTCGCAAGACCCTTCCTCT |
|  | *AmCBF1*-R | AACTCCATGTTCCTCAGCCACTCC |
| Specific primers for qRT-PCR | qA-F | GTGCTGACGTGGAATTTGATGA |
|  | qA-R | ATTTCACGGGTTGGGGTTTCT |
| Specific primers for the internal reference gene | SSU1 | AACTTAAAGGAATTGACGGAAG |
|  | SSU2 | GCATCACAGACCTGTTATTGCC |
| Primers for left side of insertion site of L28 on A05 chromosome | L-A05-1F | TGGGATTCGCCTTTGACGTT |
|  | L-A05-1R | CCTTCAACGTTGCGGTTCTG |
|  | L-A05-2F | TGGGGTTGGAGCATTAACCT |
|  | L-A05-2R | TGCGGTTCTGTCAGTTCCAA |
|  | L-A05-3F | GTCGTCGACTAAGTCTGCCT |
|  | L-A05-3R | ATGGAACGTCAGTGGAGCAT |
| Primers for right side of insertion site of L28 on A05 chromosome | R-A05-1F | GAACCTGCGTGCAATCCATC |
|  | R-A05-1R | TGTTTGGTTAAGGCGGTTGT |
|  | R-A05-4F | TGGAACGTCAGTGGAGCATT |
|  | R-A05-1R | TGTTTGGTTAAGGCGGTTGT |
|  | R-A05-6F | GATCCAGATCCGGTGCAGAT |
|  | R-A05-1R | TGTTTGGTTAAGGCGGTTGT |
| Primers for left side of insertion site of L30 on A13 chromosome | L-A13-1F | TTGCCCCTCCTAGCAATGTG |
|  | L-A13-1R | CCTTCAACGTTGCGGTTCTG |
|  | L-A13-2F | CGGACTTCGTAATTGCCCCT |
|  | L-A13-1R | CCTTCAACGTTGCGGTTCTG |
|  | L-A13-4F | AGCTCGCCACATCTGACTTT |
|  | L-A13-4R | AGATTGTCGTTTCCCGCCTT |
| Primers for right side of insertion site of L30 on A13 chromosome | R-A13-1F | ACGGTTTTTCGCCCTTTGAC |
|  | R-A13-1R | CTGGTTCTGCCTGACCATGA |
|  | R-A13-2F | CACTCAACCCTATCTCGGGC |
|  | R-A13-2R | TCGGTGCAATCATGCAACAAA |
|  | R-A13-5F | GGCCATCGCCCTGATAGAC |
|  | R-A13-5R | TGCCTGACCATGATCATTCCAA |
| Primers for the insertion site on A12 chromosome | A12-1F | TCCACACCCTGGATTTTCCG |
|  | A12-1R | GTCATGACCGGAACGAGGAG |
|  | A12-2F | AATCCAGCTGCCAAAATCGC |
|  | A12-2R | CATCATCATACCGGCAGCCA |
|  | A12-3F | AGACGCCCTTTCATAGTTGCT |
|  | A12-3R | ATTGCCGGTGGCGATTTCTA |
